# Secular Changes of Adiposity in Czech Children Aged from 3 to 6 Years: Latent Obesity in Preschool Age

**DOI:** 10.1155/2017/2478461

**Published:** 2017-11-15

**Authors:** Petr Sedlak, Jana Pařízková, Lucie Procházková, Lucie Cvrčková, Hana Dvořáková

**Affiliations:** ^1^Department of Anthropology and Human Genetics, Faculty of Science, Charles University, Vinicna 7, 128 44 Prague, Czech Republic; ^2^Department of Hygiene, Third Faculty of Medicine, Charles University, Ruska 87, 100 00 Prague, Czech Republic; ^3^Obesity Management Centre, Institute of Endocrinology, Narodni Trida 8, 116 95 Prague, Czech Republic; ^4^Faculty of Education, Charles University, M. D. Rettigove 4, 116 39 Prague, Czech Republic

## Abstract

BMI, skinfold thickness, and circumferential measures were assessed in groups of normal healthy Czech boys (*n* = 1764) and girls (*n* = 1762) 3–6 years of age in the late 1950s and 1960s (sample C), in the 1990s (sample B), and in 2014–2016 (sample A). During these decades BMI has not changed significantly, and in selected groups (boys 3, 5, and 6, girls 3 and 6 years) it was most recently found to be significantly lower (*P* ≤ 0.05). Subscapular, suprailiac, triceps, midthigh, and above patella skinfold thicknesses significantly increased in sample A as compared to sample C (*P* ≤ 0.001). Comparison of the same skinfolds measured in the nineties (sample B) and more recently (sample A) showed similar increase of subcutaneous fat (*P* ≤ 0.001). The increase of adiposity characterized by skinfolds occurring in spite of not markedly changed BMI indicates significant changes of body composition—latent (also hidden) obesity. The increase of adiposity was relatively greatest on the trunk (*P* ≤ 0.001)—which is considered a marker of the greatest health risk. The decrease of femoral circumference (*P* ≤ 0.05) along with simultaneous increase of thigh skinfold (*P* ≤ 0.01) revealed the decrease of muscle mass in the lower extremity, obviously due to the reduction of weight-transferring physical activity.

## 1. Introduction

The secular prevalence of overweight and obesity in most countries of the world has increased gradually and includes also younger, preschool children [1–4]. Latent (or also hidden) obesity, without apparently increased BMI, has been appearing more recently, that is, increased fat deposition. This concerns especially changes in body composition, simultaneously increasing body fat along with decreasing lean body mass, specifically muscles affected by hypokinesia [4, 5].

Most recent studies comparing children and adolescents with increased body weight and BMI revealed impaired motor abilities and fitness in overweight or obese subjects as compared to those with normal body weight. The results varied with regard to the character of tests which showed greater changes during activities involving the transfer of one's own body weight [6–12]. Some studies show an effect only in the obese, not overweight, children [13]. Problems of the worsening motor development of children with excessive BMI and obvious adiposity were revealed in both technically more developed and transition countries [14]. Not only the amount of fat but also its distribution can be changed manifesting increased health risks, for example, metabolic syndrome [15, 16]. This is accompanied—as a result of simultaneously reduced physical activity—by a decreased level of motor abilities and functional capacity. Cardiorespiratory fitness was also affected in early age [4, 5].

Along with increasing adiposity the secular reduction of motor abilities, strength, cardiorespiratory efficiency, and so forth was demonstrated in school children and adolescents [17, 18]. Increased BMI which can reveal excessive adiposity assessed, for example, by BIA is associated also with reduced physical activity [19, 20]. Further measurements indicated the deterioration of physical fitness in other populations.

More recent cross-sectional studies of school children and adolescents, comparing the results of adiposity changes during two-three decades (e.g., Bogalusa heart study covered the period 1972–1994) showed increase of body weight and skinfolds. This was found in schoolchildren independent of body height and other variables. Furthermore, the yearly increases in relative body weight and obesity during the latter part of this study period (1983 through 1994) were approximately fifty percent greater than those between 1973 and 1982 [21]. Garnett et al. showed that from 1985 up to 2007 more central than total adiposity (skinfolds, circumferences) has increased in Australian children [22]. Prevalence of overweight and obesity has not been reduced during the last thirty years in 3–18-year-old Polish children. Due to increasing adiposity body composition changed significantly [23].

Studies covering changes over a longer period of time (more than 50 years) with measurements of adiposity in preschool children, along with testing of motor abilities using the same methods, have been conducted in comparable Czech population. Significant secular trends for increasing fat amount and its distribution (especially on the trunk) along with decreasing motor abilities since late fifties and/or seventies of the last century up to the beginning of the twenties of this century were revealed [4, 5].

Due to increasing health problems accompanying this situation also in preschool children more measurements have been conducted. However more detailed information on further parameters of body composition (e.g., central and peripheral, external and internal fat, age at the beginning of obesity, its duration and degree, genetic factors, nutrition, and physical activity regime in the family) would be necessary. Apparently, reduced physical activity and exercise, resulting from changed lifestyle, bring about insufficient adaptation to activity and result in kinesiophobia since childhood. In addition to metabolic, cardiovascular, and so forth consequences of obesity, inadequate coordination of movements and musculoskeletal problems have been appearing, which reduce further physical activity [24]. Some of the consequences of secular change of lifestyle were therefore analyzed further.

The aim of the study was to evaluate and compare secular changes, specifically of body composition with regard to adiposity, as related to long-term changes of body weight, height, and BMI along the last five decades when significant environmental and lifestyle changes have occurred.

## 2. Materials and Methods

### 2.1. Participants

834 children aged 3 to 6 years from various kindergartens in the capital of Prague and central and southern areas of the Czech Republic during the years 2014–2016 (sample A) were followed up, *n* = 834, 413 boys, 421 girls. All children lived in a large city in comparable environmental conditions. The results of the measurements were compared with the results of the study of preschool children followed in 1990, *n* = 2,352, 1,164 boys, 1,188 girls, aged 3–6 years (sample B) [25]. This study analyzed the results compared to a representative sample of Czech population from 3 to 6 years of age in the Czech Republic. Secular changes of adiposity were evaluated also comparing the results of skinfold thicknesses measured in the fifties of the last century, *n* = 368, 181 girls, 187 boys, aged 3–5 years (sample C) [26, 27]. Also these children were from the capital of Prague and suburbs and represented an average Czech population as no significant differences with regard to urban and countryside children were found.

The Ethical Committee of the Institute of Endocrinology in Prague, Czech Republic, approved the study. The design of the study was approved by all of the participating kindergartens with the cooperation of their directors. Written informed consent was obtained from parents of all children participating in the study.

### 2.2. Measures and Procedures

The children were of middle class background; they were always followed up in their underwear during the morning session in their kindergartens, after enough sleep and more than one hour after breakfast. Their health status was always absolutely normal and children with even minor indisposition were not examined.

Anthropometry was conducted according to standard anthropometric techniques [28]. Body height was measured by portable stadiometer (Trystom; exactitude 1 mm). Body weight was ascertained using a portable personal digital scale (Tanita WB150MA S; exactitude to 0.1 kg). Circumferential measurements were conducted by soft metric tape, exactitude 1 mm. Circumference of the relaxed arm was measured at the level of the half of the distance between acromion-olecranon, at the same level where triceps skinfold was assessed. Medial circumference of the thigh was measured at the level of the distance between trochanter-tibiale, also at the level of the measurement of the thigh skinfold thickness (on the right side of the body).

Thickness of skinfolds was measured by Harpenden caliper in milimeters (for all samples), also on the right side. Results of the measurements of skinfold thicknesses in suprailiac and subscapular region, above the triceps, on the thigh and above the patella were evaluated. For the calculation of total body fat from the sum of ten skinfolds and for calculation from regression equations (according to Matiegka) a modified caliper of Best (in milimeters) for samples A and B was used, measuring on the cheek, on the neck above the hyoid bone, on chest 1 (in the anterior axillary fold along the border line of* m. pectoralis maior*), and on the chest 2 (above the 10th rib at the point of intersection with the anterior axillary line), in subscapular, abdomen, and suprailiac region, above* m. triceps *and* biceps brachii*, then also on the forearm, on the midthigh, and above patella, on calf 1 (under fossa poplitea) and calf 2 (over the line of maximal circumference) [26, 27].

Body mass index (BMI) was calculated as body weight (in kilograms)/body height^2^ (in meters). Sum of ten skinfolds was assessed according to Pařízková [26, 27]. Results of these measurements were compared with the data assessed in 1990 (sample B) in comparable groups of children as total body fat evaluated in the same manner using the formula of Matiegka [29, 30]:

(1) D=d∗S∗k2;d=12∗d1+d2+d3+d4+d5+d66.

*d*_1_ is skinfold above biceps, *d*_2_ is on the forearm, *d*_3_ is on the midthigh, *d*_4_ is on calf 2, *d*_5_ is on chest 2, *d*_6_ is on the abdomen, *S* is body surface: 71,84 *∗* body weight^0,425^*∗*body  height^0,725^, and *k*_2_ = 0.13.

The regression equation gave as a result the amount of body fat in kg and evaluated it also as the percentage of total body weight. The regression equations for the calculation of total body fat from skinfolds used in this study were derived from measurements of local Czech children [25, 29].

### 2.3. Statistical Analysis

The data for somatic traits (Sample A) were analyzed using STATISTICA software v. 9 (StatSoft, Czech Republic). The results of the measurement of sample B have been used as reference standard criteria presented as arithmetic average values mean and SD [25]. For this reason the differences between samples A and B were tested by a two-sample *t*-test.

Next we tested the secular trend of skinfold thicknesses changes assessed in the years 1957, 1959, and 1963 (sample C) compared to that in 2014–2016 (sample A) using three-way ANOVA. The effect of the following factors was considered: year of study, age, and sex. Interactions of these factors have also been evaluated. Before statistical testing, the data were transformed to follow a normal distribution. Bonferroni correction has been applied to prevent false significance. Statistical significance was assessed at the level of *P* ≤ 0.05, *P* ≤ 0.01, and *P* ≤ 0.001.

## 3. Results

Differences in body characteristics of current children (sample A) were compared to the group of Czech preschoolers measured in 1990 (sample B) which reflects the changes of economic and social conditions in the Czech Republic in the last twenty-five years (Tables 1 and 2).

Significant differences in body height were not found; children achieved comparable average values in all age categories. Significantly lower values of total body weight were found in more recent sample A in boys at the age of 3, 5, and 6 years (*P* ≤ 0.01) and in 6-year-old girls (*P* ≤ 0.05). This was confirmed also by the values of BMI which in boys were significantly lower in all age categories (*P* ≤ 0.001), excepting 4-year-old boys. In girls significantly lower values of BMI were found at the age of 3 and 6 years (*P* ≤ 0.05, *P* ≤ 0.01, resp.); on the other hand, higher values were found at the age of 5 years (*P* ≤ 0.01). At the age of 4 years no differences were revealed in girls (Tables 1 and 2).

Total body fat was evaluated by the Matiegka's and Pařízkova's method, based on local measurements of Czech children as mentioned above. Individual values of skinfolds were also evaluated on the trunk (subscapular and suprailiac) and extremities (triceps, midthigh, and patella) enabling also the evaluation of the distribution of subcutaneous fat. Changes of the circumferences of the extremities at the level of skinfolds were also evaluated, that is, circumference of the relaxed arm and medial circumference of the thigh (Tables 1 and 2).

As compared to the year 1990 (sample B) the present results (sample A) revealed significantly higher values of adiposity (*P* ≤ 0.001) in both sexes and age groups. Most marked differences in trunk (subscapular and suprailiac) and femoral regions (above patellar and midthigh skinfolds) were found (*P* ≤ 0.001). Triceps skinfold remained the same, and in 3-year-old boys triceps values were even lower (*P* ≤ 0.001).

Circumference of the arm remained the same in girls; however in 3- and 6-year-old boys it was significantly lower in current children (sample A, *P* ≤ 0.05). This finding at the age of 3 years indicates the reduction of the ratio of lean body mass, obviously muscle in this region, as triceps skinfold did not change, but the circumference of the arm decreased (*P* ≤ 0.05).

Measurements of medial circumference of the thigh revealed in current children (sample A) significantly decreased values (*P* ≤ 0.05) in 5- and 6-year-old boys along with the increase of local skinfold in 4- and 6-year-old girls (*P* ≤ 0.001). In all age groups midthigh skinfold significantly increased (*P* ≤ 0.001) without the increase of thigh circumference, which in 5-6-year-old boys decreased (*P* ≤ 0.001).

**Figure 1:**
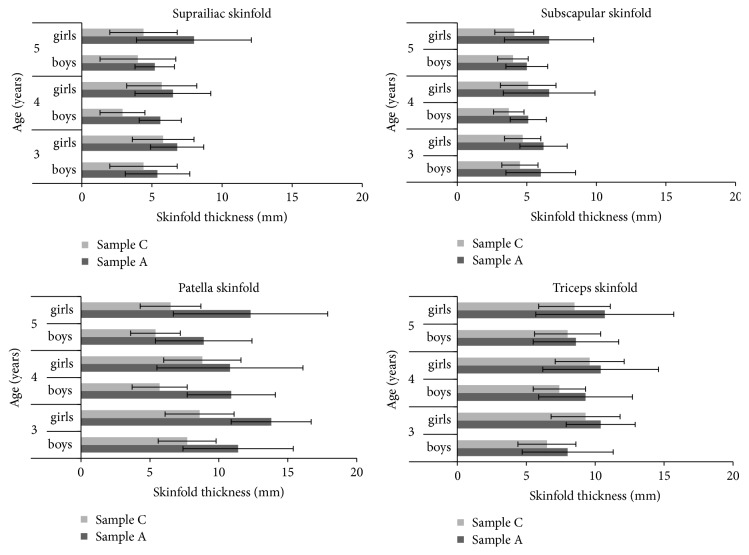
Secular changes of skinfold thickness in Czech children aged 3–5 years between the historic (C) and current (A) samples, measured by Harpenden caliper. Sample C = 1957–1963, *n* = 368 (181 girls, 187 boys); sample A = 2014–2016, *n* = 834 (421 girls, 413 boys); all differences were significant at the level *P* ≤ 0.001, based on three-way ANOVA.

The evaluation of the character of secular changes during the last 50 years—especially adiposity—was another aim of our study. The comparison of height in the group of preschoolers who were followed at the end of the fifties of the last century (sample C) with the current sample A was conducted. Girls in sample C had at the age of 3, 4, and 5 years significantly lower values of body height and weight (*P* ≤ 0.01); in boys such a difference was found only at the age of 5 years (*P* ≤ 0.05). However, the situation was opposite when BMI was compared. In current children (sample A) BMI was significantly lower, in boys in all age categories (*P* ≤ 0.01), in girls only at the age of 3 and 4 years (*P* ≤ 0.05). Results concerning adiposity were therefore quite different, triceps, thigh over patella, and subscapular and suprailiac skinfolds were found significantly higher. All mentioned comparisons confirmed significant increase of body fat in both genders and at all ages (*P* ≤ 0.001; see Figure 1) in spite of apparent overall slendering indicated by the changes of BMI.

## 4. Discussion

The present study confirmed secular increase of adiposity which was greater on the trunk [4, 18, 31, 32]. This was proved even under conditions of unchanged BMI, or a small recent decrease, indicating changes of body composition. It is possible to conclude that under conditions of increased adiposity lean especially muscle mass must have been simultaneously reduced. This also follows from the results of circumferential measurements (decreased circumference along with increased fat layer characterized by skinfold measurement), especially of the lower extremities. These are mostly influenced by weight-transferring activities which does not influence upper extremities so markedly (Tables 1 and 2). As the development of lower extremities is obviously influenced relatively much more by the reduction of overall physical activity, mentioned trend of changes in the thigh is much more obvious.

Secular changes of body composition, especially of greater adiposity focusing attention on greater deposition of fat on the trunk, concerns also preschoolers along with obvious simultaneous reduction of muscle mass, motor abilities, and physical fitness. They demonstrate therefore also increasing health risks, for example, with regard to tracking of increased weight and BMI up to primary school age [33, 34]; however, data on later longitudinal development have not been up to the present available. Moreover, children who are overweight or obese along with low level of moderate and/or vigorous physical activity in preschool age were also shown to be at greater risk of an increased systolic blood pressure, or metabolic syndrome [15, 16, 35]. Therefore, secular trend of increasing adiposity along with reduced motor development and physical fitness during early growth can be present and increase in later years [5, 8, 36].

It is necessary to emphasize that mainly studies which include not only BMI but more morphological and functional parameters assessed simultaneously in preschool children of the similar population and country, where the changes of all possible other interfering factors have been less probable, can more reliably show significant secular changes of adiposity accompanied by increasing health risks over longer periods of time [8, 34]. Decline of physical fitness resulting from reduced physical activity and exercise in recent subjects paralleled increased deposition of fat, especially on the trunk [4, 6, 18, 24, 31].

These secular changes concerning preschoolers of normal and excess weight were assessed only more recently. However, mentioned studies mostly evaluated overweight and obesity only with regard to BMI, which does not always characterize exactly the degree of total adiposity and changed fat distribution. Significant changes concerning thigh region, increase in fat layer along with the decrease of circumferential measure due to obvious reduction of muscle mass, indicate that the development of lower extremities can be influenced relatively much more by the reduction of overall physical activity, and mentioned trend of changes is therefore more apparent than in the upper extremities.

At present, this situation is mostly accompanied also by an inadequate intake of energy not corresponding to real energy needs. The composition of food intake due to the changes of overall lifestyle has not been desirable, as shown by other studies not only in Czech children [19–23, 35]. However, higher energy intake, even along with, for example, higher fats or simple carbohydrates, has been shown not to be increase BMI and adiposity, for example, in very active adolescents, participating in some organized sport training. Their functional capacity, especially cardiorespiratory efficiency, and motor abilities were found on the contrary to be significantly increased [5, 24, 37]. Food intake, significantly higher than recommended dietary allowances (RDA) and according to individual liking, does not seem to be risky in subjects who are active on the long term and have high energy expenditure.

The positive effect of adequate physical activity was shown to be effective from early childhood [5, 6, 37]. Numerous studies of lifestyle changes including reduced activity have been revealed as mostly undesirable. Therefore, the onset and causes of overweight and obesity have to be followed, analyzed, and adequately adjusted starting at a much earlier age than before. The abovementioned results on relatively greater increase of adiposity on the trunk are a special physical trait, indicating an increased risk characteristic for metabolic disorders, for example, metabolic syndrome and then cardiovascular illnesses [15, 16, 34]. This change was shown, for example, by Bogalusa Heart Study following schoolchildren from 1973 up to 1994 [21]. However, not only obvious obesity, but also the latent, “hidden” one, that is, with not markedly increased, or even slightly decreased BMI, can be a serious health risk. Young children are characterized by a natural need for high physical activity, and its reduction can have more apparent present as well as delayed effects on following development of the organism [5, 6, 24].

With regard to phylogeny, human beings have been always physically much more active which has resulted in special morphological adaptation and configuration of their organism, especially of the musculoskeletal system representing the largest part of their body. During the past decades this situation has been changing significantly due to technological development and the reduction of physical activity [38, 39]. It is necessary to emphasize that hypokinesia as a characteristic of the present lifestyle has occurred without adequate physiological adaptation during too short a period of time. This is an aggravating factor in most populations including transient and some higher social strata in developing countries [40]. All these factors along with other secular changes including inadequate nutrition have been considered for* “homo sapiens”* to be an unnatural situation especially during growth periods. The level of overall spontaneous physical activity is at this age on the highest level. Its significant reduction during this early period of life has therefore had mostly undesirable results concerning also the programming of not only present, but also future, overall development during the following periods of life. This manifests in the abovementioned somatic, metabolic, functional and so forth aspects and in addition also increases musculoskeletal problems as summarized recently (reduction of biomechanical abilities, deterioration of body posture, flat feet, back, joint and muscle pain, more frequent accidents and bone fractures, earlier arthritis, etc.) since childhood [24, 41]. This musculoskeletal situation has an essential importance as it also contributes to the further reduction of physical activity and participation in exercise, resulting in kinesiophobia which contributes to* “circulus vitiosus”* of reduced energy output and increased adiposity [41].

The results of adequate interventions and improvements of physical activity and exercise regimes as a preventive measure were reviewed recently [8, 24, 37, 40]. However, due to the life conditions, especially in large urban agglomerations, such arrangements are very difficult and require the interventions of health and pedagogic and also governmental institutions providing all groups of growing populations [34].

Interventions in physical activity regimes have shown marked positive impact [24, 37, 40]. Computer games and TV are a concern at present already preschool children who become often overly dependent on them. Suitable physical games and proper physical education and most importantly the possibility for spontaneous physical activity in an optimal and desirable environment, essential in the first place for preschoolers, should be emphasized and guaranteed [5, 8, 24]. Therefore, greater effort has to be at present undertaken to define and assure indispensable conditions and find more innovative educational approaches.

## 5. Conclusion

Comparison of basal characteristics of somatic development of preschool children—height, weight, BMI, and body composition—especially of body fat percentage and thickness of skinfolds over last five decades reveals significant differentiated secular changes. BMI does not show secular changes in preschool Czech children in spite of the case of more marked obesity that characterizes growing children in school age and adolescence. Evaluation of body composition reveals more exactly differentiated changes of somatic development along longer periods of time, aiming to disclose latent obesity already in early age. Parameters which characterize adiposity and distribution of fat as skinfolds increased significantly, especially on the trunk indicating already in preschool age possible future risks of metabolic and health disorders. Evaluation of leanness, especially muscle development of lower extremity (thigh circumference corrected with regard to skinfold, fat thickness), reveals secular decrease characterizing the impact of the reduction of physical activity level generally, due to changes of lifestyle since the beginning of life.

## Figures and Tables

**Table 1:** Comparison of monitored parameters in current Czech preschool children aged 3–6 years with survey 1990, girls.

Age (years)	3			4			5			6		
Sample	SA (*n* = 48)	SB (*n* = 286)	*P* values	SA (*n* = 123)	SB (*n* = 321)	*P* values	SA (*n* = 154)	SB (*n* = 383)	*P* values	SA (*n* = 96)	SB (*n* = 198)	*P* values
	Mean (SD)	Mean (SD)		Mean (SD)	Mean (SD)		Mean (SD)	Mean (SD)		Mean (SD)	Mean (SD)	
Height (cm)	101.0 (4.71)	100.0 (4.53)	0.123	108.2 (5.38)	107.4 (4.89)	0.134	114.5 (5.85)	113.7 (4.96)	0.110	118.7 (6.37)	119.9 (5.44)	0.095
Weight (kg)	15.5 (1.98)	15.7 (2.10)	0.539	17.5 (2.01)	17.8 (2.38)	0.216	20.6 (2.94)	20.2 (3.15)	0.176	21.4 (3.00)	22.3 (3.41)	0.028
BMI (kg/m^2^)	15.1 (1.10)	15.6 (1.40)	0.019	15.1 (1.51)	15.4 (1.50)	0.060	15.7 (1.72)	15.3 (1.50)	0.008	15.0 (1.12)	15.6 (1.80)	0.003
Body fat (%)	29.4 (3.61)	20.9 (5.35)	<0.001	23.6 (8.68)	19.8 (5.53)	<0.001	25.9 (9.81)	18.8 (6.43)	<0.001	24.9 (4.28)	18.2 (5.56)	<0.001
Sum of 10 skinfolds (mm)	84.8 (12.95)	65.2 (18.57)	<0.001	70.2 (28.33)	63.0 (19.42)	0.002	81.4 (32.75)	62.3 (26.01)	<0.001	78.8 (14.90)	59.6 (23.11)	<0.001
Subscapular skinfold (mm)^*∗*^	6.2 (1.73)	5.0 (2.05)	<0.001	6.6 (3.29)	4.8 (2.29)	<0.001	6.6 (3.16)	4.9 (3.01)	<0.001	6.4 (1.89)	4.6 (2.84)	<0.001
Suprailiac skinfold (mm)^*∗*^	6.8 (1.92)	4.2 (2.36)	<0.001	6.5 (2.69)	4.4 (2.58)	<0.001	8.0 (4.10)	4.6 (3.99)	<0.001	7.1 (1.78)	4.4 (3.20)	<0.001
Triceps skinfold (mm)^*∗*^	10.4 (2.47)	10.6 (2.86)	0.648	10.4 (4.16)	10.1 (2.98)	0.399	10.7 (5.00)	10.2 (3.33)	0.178	10.6 (2.84)	9.9 (3.27)	0.074
Mid-thigh skinfold (mm)^*∗*^	17.5 (3.13)	13.8 (4.10)	<0.001	15.4 (5.88)	13.7 (4.45)	0.001	16.8 (5.85)	13.8 (4.97)	<0.001	15.3 (3.81)	13.7 (5.10)	0.007
Patella skinfold (mm)^*∗*^	13.8 (2.87)	9.2 (3.77)	<0.001	10.8 (5.25)	8.5 (3.37)	<0.001	12.3 (5.55)	8.0 (4.00)	<0.001	11.3 (3.14)	7.2 (3.70)	0.001
Arm relax. circumf. (cm)	16.9 (0.77)	16.6 (1.22)	0.100	17.1 (1.07)	17.0 (1.33)	0.456	17.5 (3.02)	17.5 (1.54)	1.000	17.8 (1.22)	18.1 (1.52)	0.093
Thigh medial circumf. (cm)	29.8 (1.65)	29.2 (2.45)	0.103	31.5 (2.08)	30.7 (2.64)	0.003	32.0 (5.51)	32.4 (2.84)	0.271	33.1 (2.84)	33.9 (3.22)	0.039

SA = sample A: survey 2014–2016, *n* = 834 (421 girls, 413 boys); SB = sample B: survey 1990, *n* = 2,624 (1,160 girls, 1,164 boys); *∗* = measured by Harpenden caliper.

**Table 2:** Comparison of monitored parameters in current Czech preschool children aged 3–6 years with survey 1990, boys.

Age (years)	3			4			5			6		
Sample	SA (*n* = 56)	SB (*n* = 242)	*P* values	SA (*n* = 116)	SB (*n* = 326)	*P* values	SA (*n* = 152)	SB (*n* = 363)	*P* values	SA (*n* = 89)	SB (*n* = 233)	*P* values
	Mean (SD)	Mean (SD)		Mean (SD)	Mean (SD)		Mean (SD)	Mean (SD)		Mean (SD)	Mean (SD)	
Height (cm)	101.9 (4.46)	101.2 (4.96)	0.333	108.3 (3.90)	107.6 (4.65)	0.148	114.3 (4.75)	114.1 (5.20)	0.683	119.7 (5.06)	120.6 (4.72)	0.136
Weight (kg)	15.5 (1.96)	16.3 (2.00)	0.007	17.8 (2.00)	18.00 (2.22)	0.393	19.5 (2.96)	20.3 (3.22)	0.008	21.8 (3.04)	22.9 (3.29)	0.007
BMI (kg/m^2^)	14.8 (1.36)	15.8 (1.40)	<0.001	15.4 (1.32)	15.6 (1.40)	0.181	14.9 (1.16)	15.5 (1.60)	<0.001	14.7 (1.13)	15.8 (1.80)	<0.001
Body fat (%)	23.3 (8.14)	18.0 (4.34)	<0.001	23.3 (4.21)	16.7 (4.42)	<0.001	20.2 (6.20)	16.2 (5.10)	<0.001	22.1 (5.77)	15.4 (5.51)	<0.001
Sum of 10 skinfolds (mm)	67.3 (22.80)	57.6 (13.59)	<0.001	68.7 (11.70)	53.8 (14.30)	<0.001	61.6 (17.75)	53.2 (19.82)	<0.001	68.2 (15.58)	52.3 (22.10)	<0.001
Subscapular skinfold (mm)^*∗*^	6.0 (2.80)	4.4 (1.48)	<0.001	5.1 (0.28)	4.1 (1.67)	<0.001	5.0 (1.48)	4.0 (2.18)	<0.001	5.5 (1.20)	4.1 (2.30)	<0.001
Suprailiac skinfold (mm)^*∗*^	5.4 (2.33)	3.5 (1.70)	<0.001	5.6 (1.52)	3.4 (1.69)	<0.001	5.2 (1.35)	3.7 (2.90)	<0.001	6.1 (1.62)	3.8 (3.13)	<0.001
Triceps skinfold (mm)^*∗*^	8.0 (3.34)	9.4 (2.49)	0.001	9.3 (3.44)	8.8 (2.54)	0.100	8.6 (3.06)	8.8 (3.07)	0.500	8.7 (3.38)	8.2 (3.12)	0.210
Mid-thigh skinfold (mm)^*∗*^	14.0 (5.55)	11.7 (3.39	<0.001	15.1 (3.73)	11.4 (3.50)	<0.001	13.5 (5.30)	11.5 (4.23)	<0.001	14.1 (5.22)	10.8 (4.39)	<0.001
Patella skinfold (mm)^*∗*^	11.4 (3.99)	8.1 (3.09)	<0.001	10.9 (3.24)	7.0 (2.79)	<0.001	8.9 (3.50)	6.6 (2.68)	<0.001	9.3 (3.75)	6.0 (3.01)	<0.001
Arm relax. circumf. (cm)	16.3 (1.11)	16.7 (1.11)	0.016	17.1 (1.40)	17.0 (1.16)	0.452	17.0 (1.24)	17.4 (1.58)	0.006	17.2 (1.18)	18.0 (1.58)	<0.001
Thigh medial circumf. (cm)	28.3 (2.22)	28.8 (2.12)	0.117	29.6 (2.03)	29.8 (2.23)	0.397	30.4 (2.42)	31.5 (2.91)	<0.001	31.3 (2.40)	33.2 (2.64)	<0.001

SA = sample A: survey 2014–2016, *n* = 834 (421 girls, 413 boys); SB = sample B: survey 1990, *n* = 2,624 (1,160 girls, 1,164 boys); *∗* = measured by Harpenden caliper.

## References

[1] World Health Organization (2008). Obesity: preventing and managing the global epidemic. *Technical Report Series 894*.

[2] Ogden C. L., Flegal K. M., Carroll M. D., Johnson C. L. (2002). Prevalence and trends in overweight among US children and adolescents, 1999–2000. *The Journal of the American Medical Association*.

[3] Olds T. S. (2009). One million skinfolds: secular trends in the fatness of young people 1951–2004. *European Journal of Clinical Nutrition*.

[4] Sedlak P., Pařízková J., Daniš R., Dvořáková H., Vignerová J. (2015). Secular changes of adiposity and motor development in Czech preschool children: lifestyle changes in fifty-five year retrospective study. *BioMed Research International*.

[5] Pařízková J. (2010). *Nutrition, Physical Activity and Health in Early Life*.

[6] Mond J. M., Stich H., Hay P. J., Kraemer A., Baune B. T. (2007). Associations between obesity and developmental functioning in pre-school children: a population-based study. *International Journal of Obesity*.

[7] Morano M., Colella D., Caroli M. (2011). Gross motor skill performance in a sample of overweight and non-overweight preschool children. *International Journal of Pediatric Obesity*.

[8] Logan S. W., Scrabis-Fletcher K., Modlesky C., Getchell N. (2011). The relationship between motor skill proficiency and body mass index in preschool children. *Research Quarterly for Exercise and Sport*.

[9] Roberts D., Veneri D., Decker R., Gannotti M. (2012). Weight status and gross motor skill in kindergarten children. *Pediatric Physical Therapy*.

[10] Bonvin A., Barral J., Kakebeeke T. H. (2012). Weight status and gender-related differences in motor skills and in child care—based physical activity in young children. *BMC Pediatrics*.

[11] Castetbon K., Andreyeva T. (2012). Obesity and motor skills among 4 to 6-year-old children in the united states: nationally-representative surveys. *BMC Pediatrics*.

[12] Mendez-Ruiz M., Estay Carvajal J., Calzadilla Nuñez A. (2015). Comparison of psychomotor development in preschool chilean normal weight versus overweight/obesity. *Nutricio Hospitalaria*.

[13] Niederer I., Zahner L., Bürgi F. (2012). BMI group-related differences in physical fitness and physical activity in preschool-age children: a cross-sectional analysis. *Research Quarterly for Exercise and Sport*.

[14] Vameghi R., Shams A., Dehkordi P. S. S. (2012). The effect of age, sex and obesity on fundamental motor skills among 4 to 6 years-old children. *Pakistan Journal of Medical Sciences*.

[15] Elizondo-Montemayor L., Serrano-González M., Ugalde-Casas P. A., Cuello-García C., Borbolla-Escoboza J. R. (2010). Metabolic syndrome risk factors among a sample of overweight and obese Mexican children. *The Journal of Clinical Hypertension*.

[16] Maligie M., Crume T., Scherzinger A., Stamm E., Dabelea D. (2012). Adiposity, fat patterning, and the metabolic syndrome among diverse youth: the EPOCH study. *Journal of Pediatrics*.

[17] Tomkinson G. R., Olds T. S., Olds T. S. (2007). Secular changes in pediatric aerobic fitness test performance: the global picture. *Pediatric fitness. Secular Trends and Geographic Variability*.

[18] Martinez-Tellez B., Sanchez-Delgado G., Cadenas-Sanchez C. (2016). Health-related physical fitness is associated with total and central body fat in preschool children aged 3 to 5 years. *Pediatric Obesity*.

[19] Sakai T., Demura S., Fujii K. (2012). Relationship between body composition and BMI in preschool children. *Sport Sciences for Health*.

[20] Olesen L. G. R., Kristensen P. L. U., Ried-Larsen M., Grøntved A., Froberg K. (2014). Physical activity and motor skills in children attending 43 preschools: a cross-sectional study. *BMC Pediatrics*.

[21] Freedman D. S., Srinivasan S. R., Valdez R. A., Williamson D. F., Berenson G. S. (1997). Secular increases in relative weight and adiposity among children over two decades: the bogalusa heart study. *Pediatrics*.

[22] Garnett S. P., Baur L. A., Cowell C. T. (2011). The prevalence of increased central adiposity in Australian school children 1985 to 2007. *Obesity Reviews*.

[23] Kowal M., Kryst A., Woronkowicz A., Sobiecki J. (2014). Long-term changes in body composition and prevalence of overweight and obesity in girls (aged 3-18 years) from kraków (poland) from 1983, 2000 and 2010. *Annals of Human Biology*.

[24] Pařízková J. (2015). *Physical Activity, Fitness, Nutrition and Obesity during Growth*.

[25] Bláha P. (1990). *Anthropometry of Czech preschool children aged 3–7 years*.

[26] Pařízková J. (1961). Total body fat and skinfold thickness in children. *Metabolism*.

[27] Pařízková J. (1977). *Body Fat and Physical Fitness. Body Composition and Lipid Metabolism in Different Regimes of Physical Aktivity*.

[28] Eston R., Hawes M., Martin A., Reilly T., Eston R., Reilly T. (2009). Human body composition. *Kinanthropometry and Exercise Physiology Laboratory Manual*.

[29] Matiegka J. (1921). The testing of physical efficiency. *American Journal of Physical Anthropology*.

[30] Cattrysse E., Zinzen E., Caboor D., Duquet W., Van Roy P., Clarys J. P. (2002). Anthropometric fractionation of body mass: matiegka revisited. *Journal of Sports Sciences*.

[31] Pařízková J., Sedlak P., Dvořáková H. (2012). Secular trends of adiposity and motor abilities in preschool children. *Journal of Obesity & Weight Loss Therapy*.

[32] Kryst Ł., Woronkowicz A., Kowal M., Pilecki M. W., Sobiecki J. (2016). Abdominal obesity screening tools in the aspects of secular trend. *Anthropologischer Anzeiger*.

[33] Pařízková J., Heller J., Shephard R. J., Pařízková J. (1991). Relationship of dietary intake to work output and physical performance in Czechoslovak adolescents adapted to various work loads. *Human Growth, Physical Fitness and Nutrition*.

[34] Monyeki K. D., Monyeki M. A., Brits S. J., Kemper H. C. G., Makgae P. J. (2008). Development and tracking of body mass index from preschool age into adolescence in rural South African children: ellisras longitudinal growth and health study. *Journal of Health, Population and Nutrition*.

[35] Vale S., Trost S. G., Rêgo C., Abreu S., Mota J. (2015). Physical activity, obesity status, and blood pressure in preschool children. *Journal of Pediatrics*.

[36] Parizkova J. (2008). Impact of education on food behaviour, body composition and physical fitness in children. *British Journal of Nutrition*.

[37] Sigmund E., Sigmundová D., El Ansari W. (2009). Changes in physical activity in pre-schoolers and first-grade children: longitudinal study in the Czech Republic. *Child: Care, Health and Development*.

[38] Krombholz H. (2013). Motor and cognitive performance of overweight preschool children. *Perceptual and Motor Skills*.

[39] D'Hondt E., Deforche B., Gentier I. (2014). A longitudinal study of gross motor coordination and weight status in children. *Obesity*.

[40] España-Romero V., Mitchell J. A., Dowda M., O'neill J. R., Pate R. R. (2013). Objectively measured sedentary time, physical activity and markers of body fat in preschool children. *Pediatric Exercise Science*.

[41] Pařízková J., López M. Biomechanical and musculoskeletal problems in growing obese.

